# Relationships between athletic ability and academic performance in primary school students: A 3-year follow-up study

**DOI:** 10.3389/fpubh.2022.1012757

**Published:** 2023-01-20

**Authors:** Hao Zong Ji, Cui Jin Bai, Yang Yun, Wu Xiang Dong, Qian Ze Yi, Wu Dan Ping, Tang Yi, Zheng Hong Xia, Zhang Zhi Min, Xu Jing Long, Zhang Teng Fei, Wang Jun Ji, Li Zhen, Gao Zhuang, Li Ying Ke

**Affiliations:** ^1^Physical Education Teaching and Research Group, Chongqing Liangjiang Yucai Middle School, Chongqing, China; ^2^School of Physical Education, Chinese Center of Exercise Epidemiology, Northeast Normal University, Changchun, China; ^3^Physical Education Teaching and Research Group, Chengdu Caotang Primary School West Branch, Chengdu, China; ^4^English Teaching and Research Group, Chongqing Bishan Yuhu Primary School, Chongqing, China; ^5^Sports Teaching and Research Group of Xingchen School, Affiliated Middle School of West University, Chongqing, China; ^6^College of Physical Education, Yunnan University, Kunming, China; ^7^School of Physical Education, Southwest University, Chongqing, China

**Keywords:** athletic ability, academic performance, Chinese, middle school students, 3-year follow-up study, primary school students

## Abstract

**Background:**

The aim of this study was to examine whether academic performance is associated with students' athletic ability in primary school.

**Methods:**

A 3-year follow-up study was conducted among 1,136 Chinese students. Sit-up and jump rope testers were used to measure 1-min sit-ups and 1-min jump ropes, respectively. Meanwhile, the Pittsburgh Sleep Quality Scale and the Beck Depression Inventory were used to estimate sleep quality and depression levels. The end-of-semester examinations were used to evaluate students' academic performance during the follow-up period.

**Results:**

After adjusting for confounders, the mean change in Chinese language performance for participants stratified by 1-min sit-ups at baseline was 0.35 (95% CI: −0.37 to 0.76) for level 1 (slowest), 0.52 (95% CI: −0.54 to 1.08) for level 2, and 1.72 (95% CI: 1.14 to 2.30) for level 3 (fastest) (*P* for trend = 0.003); the mean change in math scores was 0.28 (95% CI: −0.50 to 0.95) for level 1 (slowest), 0.95 (95% CI: 0.38 to 1.52) for level 2, and 1.41 (95% CI: 0.82 to 1.99) for level 3 (fastest) (*P* for trend = 0.048). The mean change in foreign language scores was −0.45 (95% CI: −0.99 to −0.93) for level 1 (slowest), −0.14 (95% CI: −0.44 to 0.41) for level 2, and 0.69 (95% CI: 0.25 to 1.13) for level 3 (fastest) (*P* for trend = 0.004). The mean change in Chinese language performance for participants stratified by 1-min jump ropes at the baseline was 0.30 (95% CI: −0.16 to 0.76) for level 1 (slowest), 1.09 (95% CI: 0.42 to 1.76) for level 2, and 1.74 (95% CI: 1.14 to 2.35) for level 3 (fastest) (*P* for trend = 0.001). The mean change in math scores was 0.41 (95% CI: −0.11 to 0.92) for level 1 (slowest), 1.44 (95% CI: 0.69 to 2.19) for level 2, and 1.43 (95% CI: 0.76 to 2.10) for level 3 (fastest) (*P* for trend = 0.019). The mean change in foreign language performance was −0.71 (95% CI: −1.08 to −0.33) for level 1 (slowest), 0.95 (95% CI: −0.40 to 1.50) for level 2, and 0.91 (95% CI: 0.41 to 1.41) for level 3 (fastest) (*P* for trend < 0.001).

**Conclusion:**

This study suggests that participation in jump rope and sit-up exercises may positively affect students' academic performance.

## Introduction

Under the current education system in China, students' academic performance is the key to their access to higher education opportunities and superior educational resources, placing the question of how to effectively improve students' academic performance at the center of attention among society, schools, and families.

With the benefits of physical activity on children's physical development being universally acknowledged, a large number of foreign epidemiological surveys in recent years have also shown significant positive correlations between the amount and frequency of physical activity that the adolescent children participated in and their school behavioral performance (1–3). This correlation can be evidenced by the finding of Chaddock et al. (4) who discovered that exercise reduced activation of the prefrontal cortex and improved behavioral performance on executive control tasks in children. This association was further heightened by Han (5) study, which demonstrated that students with higher levels of physical fitness tended to have higher academic achievement. Based on many research results, experts in China, as well as in other countries, hope to help students improve their academic performance by increasing their engagement in physical activities. However, an objective and accurate evaluation of the frequency with which students participate in physical activity can be difficult to achieve in practice. Compared with frequency measurement, a comprehensive exercise capacity measurement is not only more accurate but also easier and faster to conduct and, thus, can truly reflect the daily physical activity level.

While there is no shortage of studies investigating the association between physical activity and children's behavior, most studies on the relationship between athletic ability and academic performance have been conducted in foreign countries (4, 6). The populations are mostly secondary school and college students. In the meantime, studies have yet to be conducted among Chinese students. Therefore, the object of the evaluation was to broaden the empirical base with a focus on Chinese students in primary school. Through the test of sports ability and academic performance of 1,136 third-grade primary school students in Chengdu, this study explores the relationship between sports ability and academic performance to provide theoretical support for improving primary school students' academic performance.

## Materials and methods

### Study population

The initial study was conducted in June 2019 among school children in Cheng du, China. The initial sample consisted of 1,494 pupils from primary schools, aged 10–11 (mean 10.6) years. These children underwent an athletic ability examination and completed a self-administered questionnaire; this provided information on demographics, academic performance, anthropometrics, and lifestyle factors. The same methodology was used on a follow-up, which will be explained in detail in the following paragraphs. Each child's family, contacted by phone, was informed about the study's aim and invited to participate. Meanwhile, certain participants were excluded for the following reasons:

failure to provide written informed consent for analysis of their data (*n* = 12),
missing data for the assessment (*n* = 133) or other variables (*n* = 65),
existing contraindications to exercise (*n* = 75),
body deformity (*n* = 10) at the baseline, and
missing athletic ability assessment data for 2022 (*n* = 63).

Therefore, the follow-up study included 1,136 (634 male and 502 female) participants. Ethics approval was obtained from the Institutional Review Board of the College of Physical Education of Southwest University, and written parental consent for the minors was also received before the study began.

### Measurement of athletic ability

Students' muscular endurance was assessed using 1-min rope skipping and 1-min sit-ups. The total duration of the assessment was 20 min, with rope skipping and sit-up assessment taking up 5 min and the follow-up questionnaire 15 min. The exercise ability of the investigated population was tested in June 2019, June 2021, and June 2022, respectively. The 1-min rope jumping test requires the subject to stand in the test area, start jumping rope after hearing the instructions in 3.2.1, and stop jumping rope after hearing the end instructions. The sensor would automatically record and save the test results, and this test uses the h5 tester. One-min setup requires the subject to lie on the mat, legs slightly apart, feet set on the padded instrument, knees at an approximately 90-degree angle, two fingers crossed on the back of the brain, and waist with a special electronic test belt. The participant begins to sit up when the instrument is heard to emit a “drop drop” chime. Sitting on the back must be coherent, and there is a tone. The instrument automatically counts 1. If one up and one sitting action are not in place, with no tone, then this sit-up would not count. The athlete ability of students is evaluated based on the physical health standards of Chinese students. The specific evaluation standards are as follows: During the test, we found that the number of failed students was small, and it was pointless to classify them separately for data analysis. Therefore, the passing grade and the failing grade were combined. Thus, in the subsequent data analysis, 1-min rope skipping and 1-min sit-ups were three classified variables. The evaluation criteria are shown in Tables 1–4.

**Table 1:** Single item scoring table for boys' 1-min jump rope (unit: times).

**Grade**	**Score**	**Third grade**	**Fourth grade**	**Fifth grade**	**Sixth grade**
Excellent	100	126	137	148	157
	95	121	132	143	152
	90	116	127	138	147
Good	85	110	121	132	141
	80	104	115	126	135
Pass	78	97	108	119	128
	76	90	101	112	121
	74	83	94	105	114
	72	76	87	98	107
	70	69	80	91	100
	68	62	73	84	93
	66	55	66	77	86
	64	48	59	70	79
	62	41	52	63	72
	60	34	45	56	65
Fail	50	31	42	53	62
	40	28	39	50	59
	30	25	36	47	56
	20	22	33	44	53
	10	19	30	41	50

**Table 2:** Single item scoring table for girls' 1-min jump rope (unit: times).

**Grade**	**Score**	**Third grade**	**Fourth grade**	**Fifth grade**	**Sixth grade**
Excellent	100	139	149	158	166
	95	132	142	151	159
	90	125	135	144	152
Good	85	117	127	136	144
	80	109	119	128	136
Pass	78	102	112	121	129
	76	95	105	114	122
	74	88	98	107	115
	72	81	91	100	108
	70	74	84	93	101
	68	67	77	86	94
	66	60	70	79	87
	64	53	63	72	80
	62	46	56	65	73
	60	39	49	58	66
Fail	50	36	46	55	63
	40	33	43	52	60
	30	30	40	49	57
	20	27	37	46	54
	10	24	34	43	51

**Table 3:** Scoring table of 1-min sit-ups for boys (unit: times).

**Grade**	**Score**	**Third grade**	**Fourth grade**	**Fifth grade**	**Sixth grade**
Excellent	100	48	49	50	51
	95	45	46	47	48
	90	42	43	44	45
Good	85	39	40	41	42
	80	36	37	38	39
Pass	78	34	35	36	37
	76	32	33	34	35
	74	30	31	32	33
	72	28	29	30	31
	70	26	27	28	29
	68	24	25	26	27
	66	22	23	24	25
	64	20	21	22	23
	62	18	19	20	21
	60	16	17	18	19
Fail	50	14	15	16	17
	40	12	13	14	15
	30	10	11	12	13
	20	8	9	10	11
	10	6	7	8	9

**Table 4:** Single score table of 1-min sit-ups for girls (unit: times).

**Grade**	**Score**	**Third grade**	**Fourth grade**	**Fifth grade**	**Sixth grade**
Excellent	100	46	47	48	49
	95	44	45	46	47
	90	42	43	44	45
Good	85	39	40	41	42
	80	36	37	38	39
Pass	78	34	35	36	37
	76	32	33	34	35
	74	30	31	32	33
	72	28	29	30	31
	70	26	27	28	29
	68	24	25	26	27
	66	22	23	24	25
	64	20	21	22	23
	62	18	19	20	21
	60	16	17	18	19
Fail	50	14	15	16	17
	40	12	13	14	15
	30	10	11	12	13
	20	8	9	10	11
	10	6	7	8	9

### Assessment of academic performance

This survey takes the students' Chinese, mathematics, and English scores in a 90-min examination at the end of the academic year as the core criterion for academic performance evaluation. The academic achievements of the survey population were collected in June 2019, June 2021, and June 2022, respectively. The grade was coded as follows: 4 outstanding (>90), 3 good (89–80), 2 passed (79–60), and a failure (<60).

### Confounding variables

Demographic variables and lifestyle factors were assessed using a self-administered questionnaire. Variables and factors assessed by the questionnaire included: sex (males or females), age (continuous variable), only child (yes or no), self-reported body mass index (continuous variable), parents' marital status (first marriage, divorce, or remarriage), mobile phone usage (h/day) (<1 h, 1–2 h, or >2 h), and left-behind children (which means children who grow up away from their parents) (yes or no) with established reliability and validity for assessing subjective sleep quality and quantitative sleep–wake parameters in the previous month (from self-reported measurements of sleep latency, duration, and efficiency). The answers to the 19 questions in PSQI are divided into seven parts in proportion, with a total score of 0–21. The higher the score, the worse the sleep (7). The Beck Depression Inventory (BDI) (8) was used to examine the severity of depression. It consists of 21 items. A higher score means a more severe depressive state.

### Statistical analysis

All statistical analyses were performed with SPSS software (version 20.0; SPSS, Chicago, IL, USA). For the baseline characteristics of participants, continuous variables are expressed as means and 95% confidence intervals, and categorical variables are expressed as percentages. Continuous data with skewed distributions, as determined using the Kolmogorov–Smirnov test, were logarithmically transformed. Analysis of covariance (ANCOVA) and logistic regression analysis were used to compare the baseline characteristics of the categorized group after adjustment for sex and age. One-min rope skipping and 1-min sit-ups were considered independent variables, and baseline variables were considered dependent variables. ANCOVA was also used to estimate the change in academic performance based on the categories of 1-min rope skipping and 1-min sit-ups. Model 1: adjusted for sex (categorical variable) and age (continuous variable) at the baseline. Model 2: adjusted for the variables in Model 1 and parents' educational level (categorical variable: high school degree or below and bachelor degree or above), parents' marital status (categorical variable: first marriage, divorce, or remarriage), BMI (continuous variable), only child (categorical variable: yes or no), and left-behind children (categorical variable: yes or no), at the baseline. Model 3: adjusted for the variables in Model 2 and sleep quality (continuous variable), mobile phone usage (h/day, categorical variable: <1 h, 1–2 h, and >2 h), and depression level (continuous variable) at the baseline.

## Results

The participants' baseline characteristics, according to categories of 1-min rope skipping and 1-min sit-ups, are shown in Table 5. Participants who reported higher levels of 1-min jump rope were generally younger, had lower body mass index, spent less time on their phones, and consisted of a higher proportion of males and a lower proportion of left-behind children (*P* for trend: 0.014, <0.001, 0.02, and 0.001, respectively). Other baseline characteristics did not differ significantly between categories. Similarly, participants with higher levels of 1-min sit-ups were reported to have higher proportions of males and only children, lower body mass index, better sleep quality, lower levels of depression, and less cell phone usage (P for trend: 0.05, < 0.001, <0.001, 0.018, 0.008, and <0.001, respectively). Table 6 shows the relationship between a 1-min jump rope grade at the baseline and a change in academic performance during the 3-year follow-up. After adjusting for all covariates, the higher the 1-min jump rope grade, the higher the improvement in test scores. In Model 3, the mean change in Chinese language performance in the 1-min jump rope grade was 0.30 (95% CI: −0.16 to 0.76) for level 1 (slowest), 1.09(95% CI: 0.42 to 1.76) for level 2, and 1.74 (95% CI: 1.14 to 2.35) for level 3 (fastest) (*P* for trend = 0.001); the mean change in mathematics performance in the 1-min jump rope grade was 0.41 (95% CI: −0.11 to 0.92) for level 1 (slowest), 1.44 (95% CI: 0.69 to 2.19) for level 2, and 1.43 (95% CI: 0.76 to 2.10) for level 3 (fastest) (*P* for trend = 0.019). The mean change in foreign language performance in the 1-min jump rope grade was −0.71 (95% CI: −1.08 to −0.33) for level 1 (slowest), 0.95(95% CI: −0.40 to 1.50) for level 2, and 0.91 (95% CI: 0.41 to 1.41) for level 3 (fastest) (*P* for trend <0.001).

**Table 5:** The participants' baseline characteristics, according to categories of 1-min jump rope and 1-min sit-ups.

		**One min rope skipping**				**One minute sit ups**			

**Characteristic**		**Lower** **(*****N** =* **565)**	**Middle** **(*****N** =* **341)**	**Upper** **(*****N** =* **230)**	**P for linear trend**	**Lower** **(*****N** =* **391)**	**Middle** **(*****N** =* **400)**	**Upper** **(*****N** =* **340)**	**P for linear trend**
Sex	Male	313	176	145	0.005	235	197	197	0.045
	Female	252	165	85		156	203	143	
Age (years)		10.40 (10.36, 10.44)	10.35 (10.30, 10.40)	10.1 (10.23, 10.35)	0.014	10.32 (10.27, 10.37)	10.38 (10.34, 10.43)	10.40 (10.35, 10.45)	0.064
Only child	Yes	432	235	175	0.74	235	309	298	< 0.001
	No	133	106	50		156	91	42	
Body mass index (kg/m2)		20.50 (19.88, 21.12)	19.42 (19.07, 19.77)	18.82 (18.57, 19.07)	0.000	20.97 (20.54, 21.39)	18.71 (18.43, 18.99)	18.20 (17.93, 18.47)	< 0.001
Sleep quality		8.00 (7.74, 8.26)	7.77 (7.50, 8.04)	7.66 (7.32, 7.99)	0.26	8.01 (7.73, 8.31)	7.54 (7.27, 7.80)	8.06 (7.75, 8.38)	0.018
Depression level		12.16 (11.47, 12.85)	11.92 (11.15, 12.69)	11.42 (10.50, 12.34)	0.48	12.90 (12.03, 13.76)	11.95 (11.20, 12.71)	11.12 (10.37, 11.85)	0.008
Parents marital status	First marriage	371	255	158	0.87	268	269	247	0.23
	Divorce	80	39	26		53	49	43	
	Remarriage	114	47	41		70	82	50	
Father education	High school degree or below	467	293	193	0.19	336	328	289	0.68
	Bachelor degree or above	98	48	32		55	72	51	
Mather education	High school degree or below	456	286	186	0.73	319	311	280	0.091
	Bachelor degree or above	109	73	39		72	89	60	
Mobile phone usage (h/Day)	< 1 h	413	259	189	0.02	310	313	238	0.001
	1–2 h	132	68	34		75	73	86	
	>2 h	20	14	2		16	14	6	
Left-behind children	Yes	57	35	21	0.001	42	36	35	0.841
	No	508	306	204		349	364	305	

Continuous variables without a normal distribution were log-transformed and are expressed as the estimated geometric means (95% confidence intervals). Linear trends were assessed using ANCOVA for continuous variables and logistic regression analyses for categorical variables.

**Table 6:** Multivariable-adjusted relationships of 1-min jump rope with change in academic performance during the 3-year follow-up period.

	***n =* 1,131**	**Number of participants**	**Model 2^a^**	**Model 2^b^**	**Model 3^c^**
Chinese	1 (slowest)	322	0.40 (−0.66, 0.86)	0.31 (−0.15, 0.78)	0.30 (−0.16, 0.76)
	2	260	1.09 (0.42, 1.76)	1.17 (0.50, 1.84)	1.09 (0.42, 1.76)
	3 (Fastest)	549	1.59 (0.98, 2.19)	1.67 (1.05, 2.28)	1.74 (1.14, 2.35)
	Pd		0.008	0.002	0.001
Maths	1 (slowest)	322	0.89 (−0.12, 0.90)	0.39 (−0.13, 0.89)	0.41 (−0.11, 0.92)
	2	260	1.13 (0.58, 2.06)	1.35 (0.61, 2.09)	1.44 (0.69, 2.19)
	3 (Fastest)	549	1.59 (0.92, 2.25)	1.48 (0.81, 2.15)	1.43 (0.76, 2.10)
	Pd		0.01	0.016	0.019
English	1 (slowest)	322	−0.72 (−1.09, −0.34)	−0.71 (−1.09, −0.34)	−0.71 (−1.08, −0.33)
	2	260	0.93 (0.38, 1.47)	0.92 (−0.39, 1.47)	0.95 (−0.40, 1.50)
	3 (Fastest)	549	0.93 (0.44, 1.43)	0.94 (0.44, 1.44)	0.91 (0.41, 1.41)
	P^d^		< 0.001	< 0.001	< 0.001

^a^Model 1: Adjusted for sex (categorical variable) and age (continuous variable) at the baseline. ^b^Model 2: Adjusted for the variables in model 1 and parents' educational level (categorical variable: high school degree or below and bachelor degree or above), parents' marital status (categorical variable: first marriage, divorce, or remarriage), BMI (continuous variable), only child (categorical variable: yes or no), and left-behind children (categorical variable: yes or no) at the baseline. ^c^Model 3: Adjusted for the variables in model 2 and sleep quality (continuous variable), mobile phone usage (h/Day, categorical variable: <1 h, 1–2 h, and >2 h), depression level (continuous variable) at the baseline. ^d^Linear trends were assessed using ANCOVA.

Table 7 shows the relationship between 1-min sit-ups grade at the baseline and change in academic performance during the 3-year follow-up. After adjusting for all covariates, the higher the 1-min sit-ups grade, the higher the improvement in testing score. In Model 3, the mean change in Chinese language performance in the 1-min jump rope grade was 0.35 (95% CI: −0.37 to 0.76) for level 1 (slowest), 0.52 (95% CI: −0.54 to 1.08) for level 2, and 1.72 (95% CI: 1.14 to 2.30) for level 3 (fastest) (*P* for trend = 0.003); the mean change in mathematics performance in the 1-min jump rope grade was 0.28 (95% CI: −0.50 to 0.95) for level 1 (slowest), 0.95 (0.38 to 1.52) for level 2, and 1.41 (95% CI: 0.82 to 1.99) for level 3 (fastest) (*P* for trend = 0.048). The mean change in foreign language performance in the 1-min jump rope grade was −0.45(95% CI:−0.99 to−0.93) for level 1 (slowest), −0.14 (95% CI: −0.44 to 0.41) for level 2, and 0.69 (95% CI: 0.25 to 1.13) for level 3 (fastest) (P for trend = 0.004).

**Table 7:** Multivariable-adjusted relationships of 1-min sit-ups with change in academic performance during the 3-year follow-up period.

	***n =* 1,131**	**Number of participants**	**Model 2^a^**	**Model 2^b^**	**Model 3^c^**
Chinese	1 (slowest)	276	0.38 (−0.34, 1.11)	0.29 (−0.44, 1.02)	0.35 (−0.37, 0.76)
	2	436	1.09 (−0.12, 1.13)	0.53 (−0.39, 1.10)	0.52 (−0.54, 1.08)
	3 (Fastest)	419	1.59 (1.07, 2.24)	1.75 (1.16, 2.33)	1.72 (1.14, 2.30)
	Pd		0.008	0.002	0.003
Maths	1 (slowest)	276	0.66 (−0.65, 0.78)	0.20 (−0.52, 0.93)	0.28 (−0.50, 0.95)
	2	436	0.99 (0.41, 1.55)	0.97 (0.40, 1.54)	0.95 (0.38, 1.52)
	3 (Fastest)	419	1.48 (0.89, 2.06)	1.40 (0.82, 1.99)	1.41 (0.82, 1.99)
	Pd		0.011	0.045	0.048
English	1 (slowest)	276	−0.38 (−0.91, 0.16)	−0.46 (−1.00, 0.84)	−0.45 (−0.99, −0.93)
	2	436	0.22 (−0.45, 0.40)	−0.12 (−0.44, 0.42)	−0.14 (−0.44, 0.41)
	3 (Fastest)	419	0.65 (0.21, 1.08)	0.69 (0.25, 1.13)	0.69 (0.25, 1.13)
	P^d^		0.009	0.004	0.004

^a^Model 1: Adjusted for sex (categorical variable) and age (continuous variable) (categorical variable) at the baseline. ^b^Model 2: Adjusted for the variables in model 1 and parents' educational level (categorical variable: high school degree or below and bachelor degree or above), parents' marital status (categorical variable: first marriage, divorce, or remarriage), BMI (continuous variable), only child (categorical variable: yes or no), and left–behind children (categorical variable: yes or no) at the baseline. ^c^Model 3: Adjusted for the variables in model 2 and sleep quality (continuous variable), mobile phone usage (h/Day, categorical variable: <1h, 1–2h, and >2h), and depression level (continuous variable) at the baseline. ^d^Linear trends were assessed using ANCOVA.

## Discussion

This 3-year longitudinal study examined the relationship between 1-min jump rope and sit-up grades with academic performance over time in Chinese students. These results showed that higher baseline grades of 1-min jump rope and sit-up were significantly associated with increased academic performance after adjusting for confounding factors These results showed that the better performance of these two tests at the baseline is associated with better academic achievement in the follow-up. Our findings suggest that performing 1-min jump rope and sit-up exercises has a positive effect on the academic performance of Chinese students.

The results of this longitudinal study support the hypothesis that higher levels of 1-min jump rope and 1-min sit-ups at the baseline were significantly associated with improved academic performance among Chinese children at 3 years of follow-up. The result of the study is generally consistent with the results of previous studies, such as Liao et al. (9) who found a significant positive correlation between curl scores and students' academic performance in a curl test administered to Taiwanese high school students. The result is also in line with that of Kim et al. (10) study showing that 12 weeks of skipping rope training was effective in improving the academic self-efficacy of obese female students, which in turn led to a boost in academic performance. Chen et al. (11) found that jump rope training was effective in improving cardiovascular fitness, muscular endurance, flexibility and strength, and cognitive function in a 12-week exercise intervention with obese adolescents. The 1-min rope skipping mainly tests the children's muscular endurance level, which can also reflect the students' coordination and speed quality to a certain extent. On the other hand, the 1-min sit-ups mainly test the students' abdominal flexor and hip muscle endurance levels. The results of this study infer that levels of muscular endurance may affect students' academic performance. This result is consistent with previously reported findings in children and adolescents, which suggest that adding muscular endurance training to our daily exercise routine may help to improve academic performance in elementary school students. Bass et al. (12) study found that male middle school students with higher levels of muscular endurance were 2–5 times more likely to pass math and reading tests than students with lower levels.

However, it is worth mentioning that some studies have shown no significant correlation between students' academic performance and muscle function. This may be due to differences in the age of the respondents, as well as differences in muscle function testing methods and test content. For the relationship between muscular endurance and academic performance, we infer that the reason may be due to the fact that levels of muscular endurance were in several aspects related to cognitive function, such as attention, memory, and reaction speed, a finding supported by Shigeta et al. (13). Therefore, students with higher levels of muscular endurance are normally better than students with low levels of muscular endurance, which may be one of the reasons for the difference in their academic performance; second, students with higher muscular endurance may do more muscle exercises or physical activities that require muscles in daily life. Kobilo et al. (14) found that treatment with the adenylate-activated protein kinase activator AICAR significantly enhanced the spatial memory of aged mice, whereas skeletal muscle-specific AMPKα2 subunit knockdown inhibited this effect, suggesting a strong link between the skeletal muscle and brain. Nearly 10 years of research found that the skeletal muscle is an endocrine organ that can produce and release cytokines and peptides that affect brain metabolism during muscle contraction. For example, exercise can modulate brain-derived neurotrophic by stimulating skeletal muscle BDNF (brain-derived neurotrophic factor), isrisin (Iris), interleukin-6 (IL-6) (CTSB), Exercise can modulate, for example, brain-derived neurotrophic by stimulating skeletal muscle BDNF (brain-derived neurotrophic factor), isrisin (Iris), interleukin-6 (IL-6) (CTSB), muscle growth inhibitor (MSTN), insulin-like growth factor-1 (IGF-1), vascular endothelial growth factor (VEGF), and myostatin (MSTN), level. After entering the blood circulation, the related muscle factors can cross the blood–brain barrier (BBB) and are involved in maintaining neuronal structure and function structure and function, improving synaptic plasticity, reducing central inflammation, and increasing cerebral angiogenesis. Brain-derived neurotrophic factor (BDNF) is a member of the neurotrophic factor family and is currently the most studied neurotrophic factor involved in the regulation of adult neurogenesis and synaptic plasticity muscle factors involved in the regulation of neurogenesis and synaptic plasticity in adults. Studies have shown that circulating BDNF crosses the blood–brain barrier and can stimulate the production of the hippocampus to stimulate central trophic factor production and improve short-term cognitive performance and long-term morphological adaptation in the brain. Longitudinal studies have shown that during exercise, skeletal muscle secretes a transcriptional co-activator called PGC-1α of which FNDC5 is a downstream protein. During exercise, FNDC5 is broken down into irisin in the exercising muscle (15). Irisin is associated with neural differentiation, crosses the blood–brain barrier, acts on endothelial cell receptors or brain BDNF receptors, and directly regulates hippocampal gene expression. Brain-derived neurotrophic factor (BDNF) promotes neurotic proliferation, growth, differentiation, and synaptic regeneration, which are key functions related to learning, memory, and biological regeneration (16). Thus, frequent muscle activity may improve brain metabolism by enhancing the production of cytokines and peptides. This study shows that regular participation in rope skipping and sit-ups can effectively improve the academic performance of primary school students.

One limitation of this study, inherited from previous morpheme studies, is that the subjects of this survey are primary school students in Chengdu, which means that the findings are not wholly generalizable to students in other areas and whether the research results can be applied to other regions and populations need further research. Second, although the survey is conducted through the authoritative scale, the subjective factors of participants may still affect the research results.

## Conclusion

In conclusion, after adjusting many covariates, this study found that higher levels of 1-min jump rope and 1-min sit-ups at the baseline were significantly associated with improved academic performance among Chinese children at 3 years of follow-up. This study suggests that participation in jump rope and sit-up exercises may positively affect the improvement of students' academic performance. Further intervention studies are needed to explore the causality between 1-min jump rope and sit-up grades with academic performance.

## Data availability statement

The original contributions presented in the study are included in the article/supplementary files, further inquiries can be directed to the corresponding author.

## Ethics statement

Ethics approval was obtained from the Institutional Review Board of the College of Physical Education of Southwest University. Written informed consent to participate in this study was provided by the participants' legal guardian/next of kin.

## Author contributions

HJ, CB, and YY have given substantial contributions to the conception or the design of the manuscript. QY, WP, ZM, ZX, GZ, XL, ZF, WJ, and LK contributed to the acquisition, analysis and interpretation of the data. TY made an important contribution to the polishing of the manuscript. HJ had critically revised the manuscript. All authors participated to drafting the manuscript, read, and approved the final version of the manuscript.
